# Concurrent Word Generation and Motor Performance: Further Evidence for Language-Motor Interaction

**DOI:** 10.1371/journal.pone.0037094

**Published:** 2012-05-15

**Authors:** Amy D. Rodriguez, Matthew L. McCabe, Joe R. Nocera, Jamie Reilly

**Affiliations:** 1 Department of Speech, Language, and Hearing Sciences, University of Florida, Gainesville, Florida, United States of America; 2 Brain Rehabilitation Research Center, Malcom Randall VA Medical Center (VAMC), Gainesville, Florida, United States of America; 3 Schwob School of Music, Columbus State University, Columbus, Georgia, United States of America; 4 Department of Aging & Geriatric Research, University of Florida, Gainesville, Florida, United States of America; Royal Holloway - University of London, United Kingdom

## Abstract

Embodied/modality-specific theories of semantic memory propose that sensorimotor representations play an important role in perception and action. A large body of evidence supports the notion that concepts involving human motor action (i.e., semantic-motor representations) are processed in both language and motor regions of the brain. However, most studies have focused on perceptual tasks, leaving unanswered questions about language-motor interaction during production tasks. Thus, we investigated the effects of shared semantic-motor representations on concurrent language and motor production tasks in healthy young adults, manipulating the semantic task (motor-related vs. nonmotor-related words) and the motor task (i.e., standing still and finger-tapping). In Experiment 1 (n = 20), we demonstrated that motor-related word generation was sufficient to affect postural control. In Experiment 2 (n = 40), we demonstrated that motor-related word generation was sufficient to facilitate word generation and finger tapping. We conclude that engaging semantic-motor representations can have a reciprocal influence on motor and language production. Our study provides additional support for functional language-motor interaction, as well as embodied/modality-specific theories.

## Introduction

Semantic memory is a subsystem of human memory that underlies knowledge of word and object meaning. As such, this form of memory acts as the substrate for many of our most fundamental interactions with the world. Our understanding of semantic memory has rapidly evolved during the last few decades. A dominant prior approach to semantic memory with roots in philosophy held that humans represent object knowledge via an abstract, amodal manner that does not honor the sensorimotor features of objects [1]. This disembodied view of conceptual knowledge has waned in favor of theories premised upon modality-specific roles of perception, action, and mental simulation [2]. Most contemporary theories of semantic memory exist along a continuum from abstract propositional (or modality-neutral) to embodied (or modality-specific). Today, a condition of virtually all neurologically constrained theories of semantic memory is that they must specify the extent to which object concepts are grounded in perception and action.

The strictest account of embodied cognition holds that object concepts are represented as fully distributed patterns of activation across modality-specific brain regions that are also engaged during actual perception or use [3]. For example, a word such as ‘writing’ cannot be understood without supportive perceptual enactment by corresponding regions of premotor and supplementary motor cortex [3]. Under this view, perceptual and conceptual processes engage the same neural architecture and are thus inextricably linked.

One of the principal criticisms of embodied cognition is that proponents have offered few satisfactory explanations for how abstract concepts (i.e., truth, empathy) might *necessarily* be represented by sensorimotor features [4]. As a consequence, more moderate embodiment approaches have emerged which are premised upon the idea that perceptual processes are *sufficient* to engage semantic processing, an effect that is most evident when there is a high degree of correspondence between an action word and its associated gesture or body part (e.g., pushing a button with one's hand in response to a hand-related word). In the current study, we examine such an approach to semantic representation, with a focus on investigating the interaction between language and motor production. More specifically, we investigate the dual task effects associated with producing motor-related words while simultaneously engaged in motor production tasks.

### Evidence for Functional Language-Motor Interaction

Pulvermüller and colleagues have perhaps presented the most compelling body of research in support of the hypothesis that motor words and motor actions share some degree of somatotopic cortical representation [5]. For example, numerous neurophysiological studies have demonstrated that perception of action words almost simultaneously elicits activation in regions adjacent to the primary motor cortex. This phenomenon has been observed during silent word reading [6], [7] and when making auditory lexical decisions for words with high degree of motor salience [8], [9]. Other work from Vigliocco and colleagues has demonstrated that implicit auditory comprehension results in a frontal-temporal dissociation for motor-related words (e.g., walks) relative to sensory words (e.g., darkness) [10]. In summary, there is a large body of evidence to support activation of motor regions in language perception and comprehension tasks.

Considerably fewer studies have investigated language-motor interactions during production tasks; however, a number of functional magnetic resonance imaging (fMRI) investigating sequential language-motor experiments are noteworthy exceptions. Vitali and colleagues [11], for example, contrasted the BOLD response while participants produced tool names relative to animal names. The tools vs. animals contrast revealed peaks in inferior prefrontal and premotor cortex, regions critical for motor planning and motor imagery. Similar patterns of activation were revealed by Esopenko and colleagues [12] during an action word association verification task (e.g., “pencil” for “writing”), and Oliveri and colleagues [13] during overt production of action words. Recently, Peran and colleagues [14] also found left prefrontal and premotor activation during generation of action words representing different classes of manipulable objects (e.g., screwdriver). In a series of behavioral experiments, Morsella [15] investigated the language-motor interaction during word production, but little remains known about concurrent language-motor effects.

Our aim was to extend the incipient body of literature on language-motor interaction during concurrent production tasks within the framework of two hypotheses: 1) Semantic representations of action-related words are functionally linked to the motor system. That is, motor-related words activate neuroanatomical structures in the motor system during meaningful processing [16]; and 2) Similar functional networks support aspects of language and motor production. That is, words and actions have shared neuroanatomical underpinnings that support both cognitive and motor processes [16]. We investigated the effects of shared semantic-motor representations on concurrent language and motor production tasks in two orthogonal experiments. Word generation was paired with a gross motor production task (i.e., postural control) and fine motor production task (i.e., finger tapping). The rationale for using two different methods was to investigate the effect of word meaning on different types of bodily activity. By measuring postural control, we aimed to show that word meaning can affect involuntary, static motor performance at the body level. By measuring finger tapping, we aimed to show that word meaning can affect self-initiated, dynamic motor performance of a specific body part. We tested the following predictions:

1. Production of motor-related words (relative to nonmotor words) will differentially affect gross motor activity (i.e., postural control). Due to the overlap in brain areas that underlie semantic-motor representations, motor-related words will facilitate movement, thereby interfering with (or inhibiting) postural stability.
2. Production of motor-related words (relative to nonmotor words) will differentially affect fine motor activity (i.e., finger tapping). Due to the overlap in brain areas that underlie semantic-motor representations, motor-related words will facilitate motor production.

Previous studies have investigated the effects of concurrent motor and language production tasks (e.g., walking and talking, tapping and talking) from the standpoint of dual task interference, focusing specifically on the effects of increased attentional demands and cognitive complexity on decrements in performance [17], [18]. Our approach is different in that we focused on the effect of semantic content on language-motor interaction.

## Experiment 1

We examined the center of pressure (COP) displacement while participants stood on a balance board and performed a number of verbal fluency tasks (i.e., generating words from a given semantic category such as animals or tools). The COP trajectory is quantified in upright stance by measuring forces exerted against the ground at the location of the COP as the body attempts to maintain the center of mass within the base of support [19], [20]. Alterations in the displacement of the COP are sensitive to changes in balance coordination [20]. In fact, studies have demonstrated that COP displacement is valid and reliable for understanding postural control in both patient and healthy populations [20], [21].

During the verbal fluency tasks, participants generated words from semantic categories with either high human motor salience or whose meaning carried little to no motor salience. The independent linguistic variable was the semantic nature of the verbal fluency category, and the dependent variable was distribution the COP.

### Ethics Statement

This study was approved by the Institutional Review Board at the University of Florida. All participants provided written informed consent prior to participation. Ethical standards were followed in the conduct of this research.

### Participants

Participants included 23 healthy young adults (n = 7 males, n = 16 females) recruited from the University of Florida who were right-handed, native English speakers and free of cognitive or motor impairment. Mean age was 20.8 (range 18–26 years).

### Procedure and Materials

Testing was completed in a quiet laboratory equipped with a Wii Balance Board (WiiBB) (Nintendo, Kyoto, Japan) to capture changes in COP and an ambient microphone to record each participant's word production. The WiiBB was recently demonstrated as a valid and reliable tool for assessing postural control via COP displacement [22]. Data from the WiiBB and microphone were recorded to a Macbook Pro computer to separate, synchronized channels. Participants removed their shoes and stood with their feet equally spaced across the surface of the WiiBB. The participants then completed a series of tasks while we monitored their postural control using COP measurements.

First, we administered two 60-second baseline motor conditions. In the first of these conditions, participants stood silently while we recorded the displacement of the COP. This condition was implemented to assess variability in postural control in the absence of additional speech, motor, or cognitive demands. For the second baseline condition, we measured distributions of COP while participants counted aloud as quickly and steadily as possible (i.e., one, one-thousand, two, one-thousand, three, one-thousand,,,). This condition allowed us to assess the effect of concurrent speech on postural control while minimizing extraneous cognitive demands, as we considered counting an automatic task that is minimally taxing on cognitive abilities such as working memory or lexical retrieval.

Upon completion of the baseline conditions, participants completed a series of concurrent language-motor tasks (i.e., verbal fluency) while we captured their COP displacement. For these verbal fluency conditions, participants were cued with a semantic category (e.g., animals) and asked to name as many different items belonging to that particular category as possible while standing as steadily as possible and maintaining visual attention on a fixation point positioned approximately 3 feet away. Following each category cue, participants heard a pure tone “beep” cueing them to begin. We halted production after 60 seconds and proceeded to the next semantic category.

The experiment comprised six verbal fluency categories that were either associated with human motor action (i.e., Semantic-Motor categories) or nonmotor in nature (i.e., Semantic-Other categories). The difference in these two categories is the degree to which exemplars more strongly defined by motor or visual features. The exemplars in the Semantic-Motor categories have more salient motor-related features, while the exemplars in the Semantic-Other categories have more salient visually-related features. This categorically-based comparison of motor vs. visual words is commonly used in studies investigating language-motor system interaction. The Semantic-Motor categories were 1) musical instruments; 2) garage tools; and 3) school/office supplies. These three categories were considered Semantic-Motor due to the requirement of human motor action/manipulation to fulfill the function of the corresponding exemplars (i.e., garage tools>hammer>to use a hammer you must grasp it with the hand). The Semantic-Other categories were 1) animals; 2) cities; and 3) fruits and vegetables. These three categories were considered Semantic-Other due to form and visual features of the corresponding exemplars (i.e., fruits and vegetables>orange>it is round and orange in color). Category presentation was fully randomized across participants.

### Data Acquisition and Coding

To acquire COP data during the Baseline Motor and Motor+Language tasks, we used the WiiBB and a software interface, OS*Culator* (Trolliard, 2010), which routed incoming Bluetooth wireless data to Cycling ‘74's Max5 software (2009). Data were acquired at 100 ms intervals from each of the four pressure sensors on the WiiBB. This sampling rate provided not only sufficient data for analysis, but also a 10 Hz low-pass filter on the data streams to account for high-frequency sensor jitter that is characteristic of piezoelectric force plates [22], [23], [24].

Mean COP distribution was calculated for the Baseline Motor conditions and Motor+Language conditions using real-time data from each of the four WiBB sensors. Based on these raw pressure sensor data, we derived X,Y coordinates for each person's COP at each time point using the following equation:
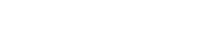


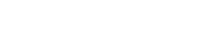
Note: TL = Top Left Sensor, TR = Top Right Sensor, BL = Bottom Left Sensor, BR = Bottom Right Sensor

Since the WiiBB corresponds to a two-dimensional Cartesian coordinate system, point-to-point measures of Euclidean distance were derived to calculate the absolute value of COP distance traveled from each successive time point. For this distance estimation, we used the standard linear distance formula for a Cartesian plane:
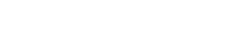



The COP data, obtained in discrete intervals of 100 ms, yielded 600 data points for each 60 s verbal fluency condition. Simultaneous to collecting the COP data, we digitally recorded verbal responses. The verbal output data, which was time-locked in 100 ms epochs was categorically coded as either 1) overt speaking; 2) semantic processing and motor initiation 500 ms preceding speech onset; or 3) silence. We eliminated epochs corresponding to complete silence and focused our analyses on the speaking and semantic processing conditions.

For the statistical analyses to follow we derived a word-COP displacement ratio by dividing the average amount of COP displacement by the total number of words produced in each of the verbal fluency categories. For example, a participant in the animal naming condition who named 20 animals in one minute and displaced their COP an average of .0036 in distance would have a COP displacement ratio of .0036/20 (or .00018). Then we averaged these ratios across the two semantic conditions. This ratio conversion allowed us to contrast the two conditions using a common scale, which accounted for differences in the number of exemplars produced.

### Results

A comparison of the baseline conditions revealed that participants COP displacement was increased by a factor of 1.75 in the counting relative to the standing silent condition [paired *t*(22) = 3.27, *p* = .004, *d* = .61]. This suggests that the act of speaking significantly modulates one's postural control when additional cognitive demands are minimized through performing a simple counting task.

Regarding the experimental conditions, there was also a significant difference in COP displacement when participants produced Semantic-Motor category exemplars relative to Semantic-Other categories [paired *t*(22) = 3.39, *p* = .003, *d* = .48]. That is, participants increased their COP displacement by a factor of 1.2 times more when producing words with high motor salience relative to non-motor related words.

**Figure 1:**
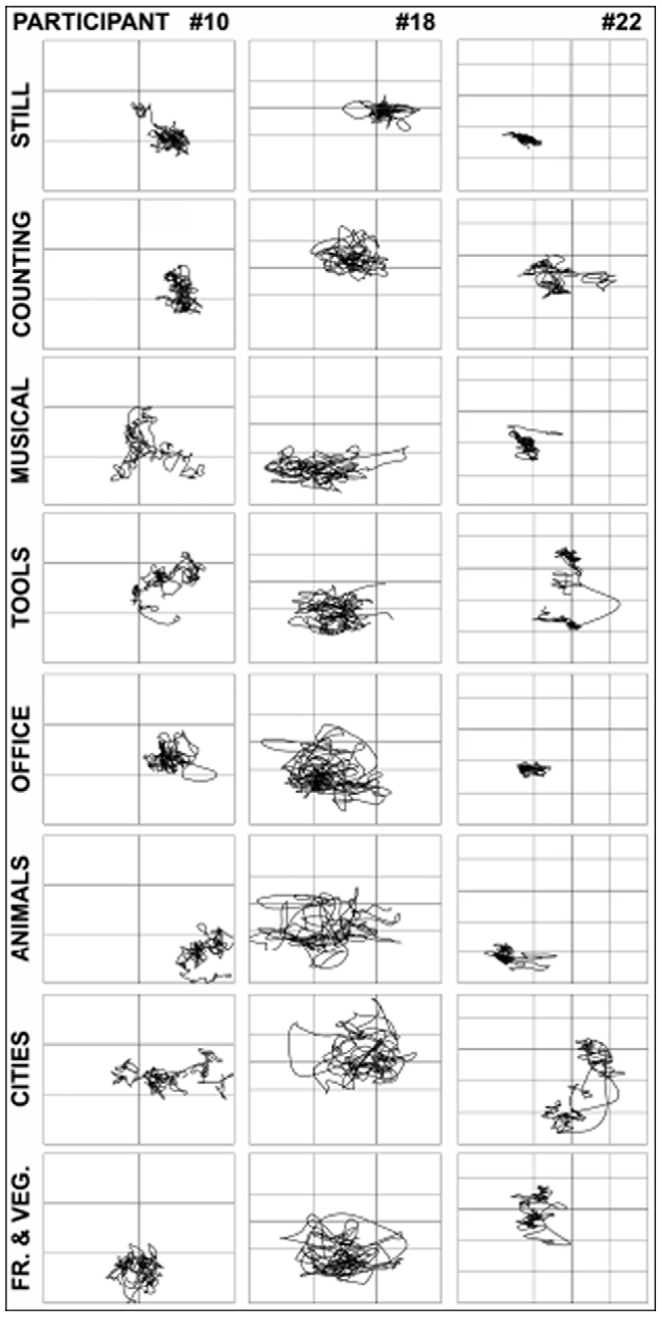
Depiction of COP displacement by semantic condition. Note: This figure shows center of pressure (COP) displacements for three selected participants. As described in detail in the method section, the COP displacement is the absolute value of the distance traveled every 100 ms of the COP during each of the eight conditions (i.e., silent standing, Baseline Motor condition, Semantic-Motor conditions and Semantic-Other conditions). Note the larger displacement area within each participant during the Semantic-Motor conditions when compared to the Semantic-Other conditions.

**Figure 2:**
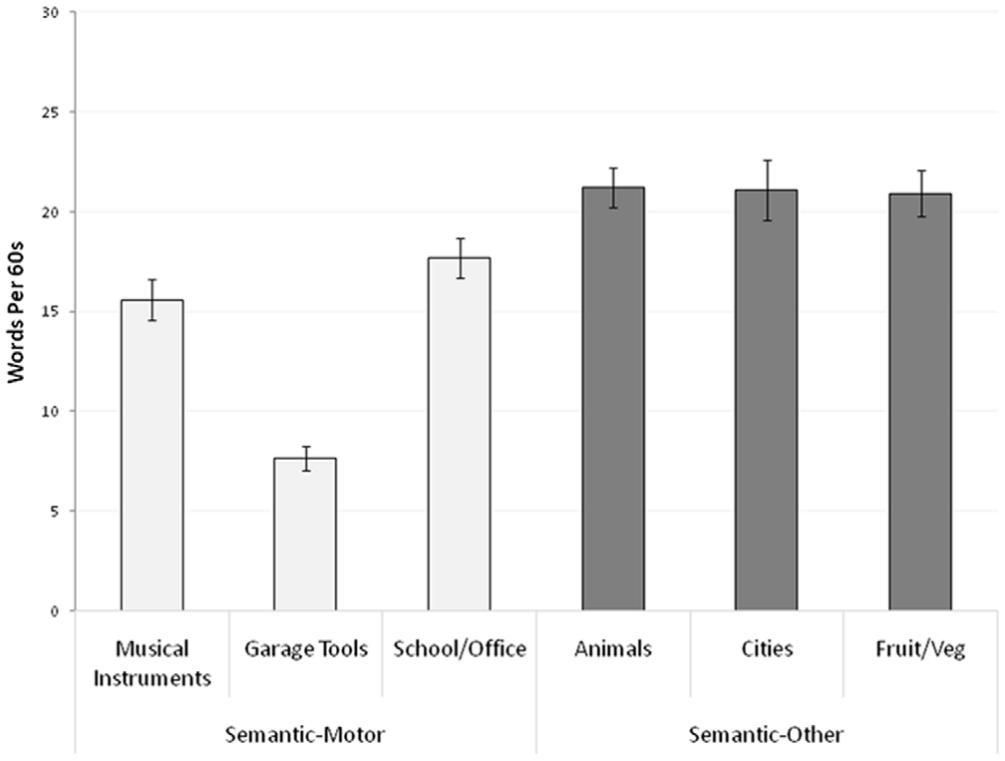
Depiction of word production means by semantic condition.

COP displacement distribution means appear in Table 1. Additionally, a graphic depiction of COP displacement for three participants in each condition (i.e., Silent, Counting, and all six semantic categories) appears in Figure 1. Word production means and word duration means for each semantic category appear in Figure 2 and Table 2, respectively.

**Table 1:** COP Displacement Means by Semantic Condition.

	COP Displacement	
	Mean	SD
**Baseline**		
Silent	.0080	.007
Counting	.0143	.013
**Semantic-Motor**		
Musical instruments	.0166	.021
Garage tools	.0153	.017
School and office supplies	.0141	.013
*Total Semantic-Motor*	.0153	.017
**Semantic-Other**		
Animals	.0152	.016
Cities	.0139	.012
Fruits and Vegetables	.0137	.014
*Total Semantic-Other*	.0143	.014

**Table 2:** Word Duration Means by Semantic Condition (Exp 1).

	Mean	SD
**Semantic-Motor**		
Musical instruments	.844	.149
Garage tools	.865	.161
School and office supplies	.869	.143
*Total Semantic-Motor*	.859	.009
**Semantic-Other**		
Animals	.792	.114
Cities	.909	.156
Fruits and Vegetables	.877	.129
*Total Semantic-Other*	.859	.021

Participants produced more words in the Semantic-Other categories relative to the Semantic Motor categories [mean Semantic-Motor: 13.5; mean Semantic-Other: 21.1, paired *t*(22) = 9.09, p<.001, *d* = 1.93] but mean word duration across semantic conditions was not significantly different (p = . 979). This higher rate of speech productivity in the Semantic-Other condition introduces a potential confound due to the possibility that differences in COP displacement is the result of differences in the amount of concurrent motor activity between the two semantic conditions. In order to evaluate this possibility we analyzed performance relative to each participant's counting performance. The average amount of COP displacement in the counting condition did not significantly differ from the Semantic-Motor condition [paired *t*(22) = 1.00, *p* = .33, *d* = .07] or the Semantic-Other condition [paired *t*(22) = .02, *p* = .98, *d* = .001], indicating that the simple effect of more speaking cannot entirely account for these differences.

Additionally, it could be argued that participants produced fewer words in the Semantic-Motor condition because the categories were more difficult (i.e., higher cognitive load). To investigate this possibility, we conducted an analysis of covariance (ANCOVA) with semantic condition as a two-level within-subjects factor and COP as the dependent variable. A difference score, derived by subtracting number of words produced in the Semantic-Other condition from number of words produced in the Semantic-Motor condition, was the covariate. The ANCOVA revealed no significant difference between the two semantic conditions [*F*(1,21) = .005, *p* = .102]. As such, it appears that increased cognitive load in the Semantic-Motor condition may have contributed to our finding. However, two observations are worth noting. First, the word production mean for the Semantic-Motor category “garage tools” was significantly lower (7.65) than the musical instruments and school/office supplies (15.57 and 17.17, respectively). Decreased word production in this category may have skewed the results due to less activation of the motor system. Secondly, the p-value suggests there is a trend in the data toward an effect of motor salience on COP displacement. Perhaps inclusion of different categories that elicit production of a greater number of motor-related words would have yielded a significant effect.

### Interim Discussion

Production of motor-related words while standing increased COP displacement. The finding that COP displacement was not significantly different between the counting condition and either of the semantic conditions precludes an explanation based on amount of concurrent motor activity. While increased cognitive load may have contributed to our findings, the interpretation that higher cognitive load increased COP displacement in the Semantic-Motor condition is based on the assumption that number of words produced is an index of increased cognitive complexity. From our point of view, this remains an empirical question. Additionally, a trend for the effect of motor salience on COP displacement was noted. Thus, we find some support for our hypothesis that motor-related words would facilitate movement and therefore inhibit postural stability. We propose that this is at least in part due to the shared neuroanatomical structures that support underlying motoric aspects of motor-related word production and postural control. That is, the content of what is said has a direct and rapid influence on the motor system at a body level when semantic-motor representations are engaged.

## Experiment 2

We examined effects of the semantic content of word production on performing a concurrent fine motor task. More specifically, we analyzed dual task effects of finger tapping and verbal fluency. The independent linguistic variable was semantic nature of the verbal fluency category (i.e., human motor vs. nonmotor). Rates of finger tapping and word production were treated as dependent variables.

### Ethics Statement

This study was approved by the Institutional Review Board at the University of Florida. All participants provided written informed consent prior to participation. Ethical standards were followed in the conduct of this research.

### Participants

Forty healthy young adults (n = 5 males, n = 35 females) were recruited from the University of Florida who did not participate in Experiment 1(see Experiment 1 for additional exclusion criteria). Mean age was 19.6 (range = 18–22 years).

### Procedure and Materials

Experiment 2 was conducted on a desktop computer equipped with EPrime 2.0 stimulus delivery software and coupled to a serial response (SR) button box, as well as a headset microphone coupled to a digital recorder. Participants were fitted with the headset microphone and seated in a quiet laboratory with the SR button box positioned within comfortable reach of their hand. They were instructed to tap the SR button with their index finger as quickly as possible, while simultaneously generating exemplars to categories as quickly and accurately as possible. In order to assess possible interference effects from left/right hemisphere demands on finger tapping responses and speech, we counterbalanced response hand by having half of the participants tap with their left index finger and half tap with their right index finger.

Experiment 2 comprised one Baseline Motor condition, two Baseline Language conditions, and four Motor+Language conditions. Each condition was 60 seconds in duration, paced by cues from the E-Prime program. In the Baseline Motor condition participants remained silent while tapping the SR button for 60 seconds.

In the Baseline Language condition participants produced exemplars for one Semantic-Motor category and one Semantic-Other category (no finger tapping).

In the Motor+Language condition participants produced category exemplars while tapping finger tapping (n = 2 Semantic-Motor categories, n = 2 Semantic-Other categories).

In Experiment 2, the Semantic-Motor categories were: a) things you do with your hands; b) objects that require the use of your hands; and c) musical instruments. Thus, two of the three Semantic-Motor categories differed from Experiments 1. This change was implemented in an effort to increase the number of words generated and exploit the motor salience variable. As with Experiment 1, the Semantic-Motor categories were contingent on the requirement of human motor action/manipulation to fulfill the function of the corresponding exemplars (as in the categories “musical instruments” and “objects that require the use of your hands”) or to complete the named task (as in the category “things you do with your hands”). While the latter two categories are more ad-hoc in nature, selection was based on their exemplars' motor salience. The Semantic-Other categories did not differ from Experiment 1: (i.e., animals, cities; fruits/vegetables). The semantic categories used in the Baseline Language and Motor+Language conditions were randomized across participants.

### Data Acquisition and Coding

The SR box sampled finger tapping rate in 100 ms epochs, resulting in 600 data points per category. The verbal responses were digitally recorded and time-locked to the tapping data. The epochs were coded using the same procedure described in Experiment 1. We then derived numerical values for finger tapping and word production for Time on Task (i.e., time spent tapping and speaking only, silences excluded). The resulting values were used to derive the following finger-tapping and word production proportions reflecting concurrent (i.e., dual task) performance:

- Finger tapping: Mean number of taps for speaking time during the Motor+Language conditions divided by the mean number of taps during 60 second Baseline Motor condition
- Word production: Mean number of words for speaking time during the Motor+Language conditions divided by the mean number of words during speaking time for Baseline Language condition

### Results

Participants showed no right-left hand advantage for the Semantic-Motor or Semantic-Other conditions, so we collapsed the handedness factor and then conducted a 2*2 repeated measures multivariate analysis of variance (MANOVA). The first factor was Condition (i.e., Semantic-Motor and Semantic-Other); the second factor was Task (i.e., finger tapping and word production).

**Figure 3:**
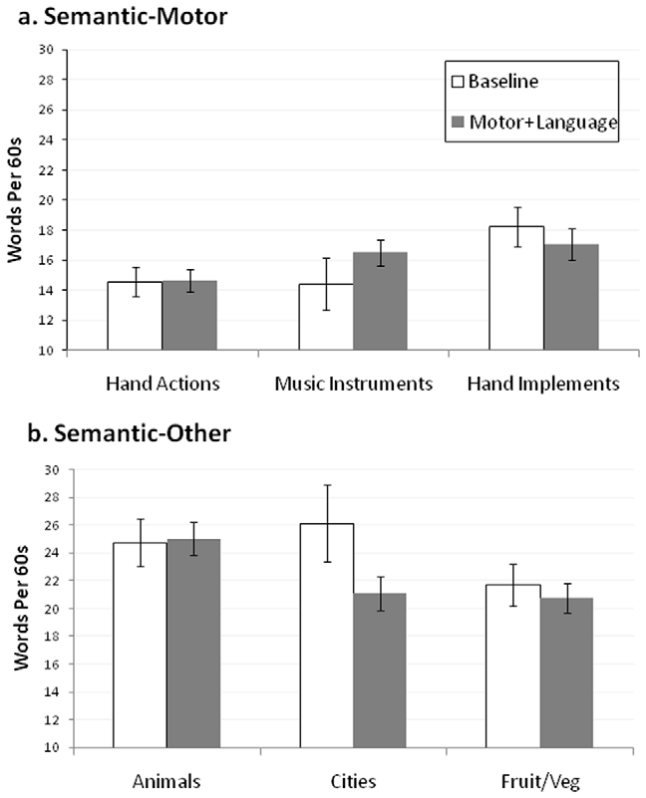
Depiction of word production means during Baseline and Motor+Language conditions for Semantic-Motor and Semantic-Other categories. Note: “Hand Actions” refers to the category “Things you do with your hands”. “Hand Implements” refers to the category “Objects that require the use of your hands”.

The MANOVA revealed a significant main effect of Condition [F(1, 39) = 5.18, *p* = .03, partial η^2^ = .12], indicating that dual task effects in the Semantic-Other condition were greater than dual task effects in the Semantic-Motor condition. In contrast, there was no main effect of Task, nor was there a Condition*Task interaction. Finger tapping data are reported in Table 3. A comparison of word production means for Baseline and Motor+Language conditions are depicted for Semantic-Motor and Semantic-Other categories in Figure 3. Additionally, mean word duration across categories, which did not significantly differ between semantic conditions (p = .242), is reported in Table 4.

**Table 3:** Finger Tapping Means by Semantic Condition.

	Baseline		Motor+Language	
	Mean	SD	Mean	SD
	218.93	35.72		
**Semantic-Motor**				
Things you do with your hands			213.04	43.27
Musical instruments			206.60	41.59
Objects that require use of your hands			213.83	30.68
*Total Semantic-Motor*			210.86	38.51
**Semantic-Other**				
Animals			191.96	48.34
Cities			206.15	30.83
Fruits and Vegetables			206.81	51.74
*Total Semantic-Other*			201.40	39.28

**Table 4:** Word Duration Means by Semantic Condition (Exp 2).

	Mean	SD
**Semantic-Motor**		
Things you do with your hands	.881	.129
Musical instruments	.938	.119
Objects that require use of your hands	.912	.124
*Total Semantic-Motor*	.910	.005
**Semantic-Other**		
Animals	.852	.098
Cities	1.00	.241
Fruits and Vegetables	.939	.128
*Total Semantic-Other*	.932	.075

Since there was a main effect of Condition, we conducted a one sample post hoc t-test to further explore task performance in each semantic condition. We compared finger tapping and word production values to a value of one (or baseline performance) to determine if there was a significant dual task effect. Neither tapping nor word production significantly differed from baseline in the Semantic-Motor condition. In contrast, the Semantic-Other condition revealed a marginally significant difference from baseline performance for tapping [*t*(39) = 2.50, *p* = .02, *d* = .79] and a significant difference from baseline performance for word production [*t*(39) = 3.83, *p*<.0001, *d* = 1.23], indicating a reduction in both finger tapping and word production performance in this condition. As such, we observed a greater effect of dual task in the Semantic-Other condition relative to the Semantic-Motor condition for both tasks.

### Interim Discussion

Concurrent finger tapping and word generation interfered with finger tapping and significantly slowed word production in the Semantic-Other condition, a finding that is consistent with the dual task literature. However, tapping performance and word production in the Semantic-Motor condition was preserved (no significant change from baseline). As it is well established that performance on one or both tasks suffers under dual-task conditions, we interpret the lack of change between Baseline and Semantic-Motor conditions as facilitation of production.

There are several possible explanations for these observed effects that are not explicitly rooted in a higher-level embodied account of language and action. For instance, it is possible that participants spontaneously prioritized the cognitive task in the Semantic-Other condition, resulting in diminished finger tapping rate. Another alternative account holds that producing a larger number of words in the Semantic-Other condition relative to the Semantic-Motor condition resulted in the significant decrease in finger tapping in the former.

There are several reasons to conclude that these alternate accounts do not offer an exhaustive explanation of the data. The first is that there was a significant decrease in time spent producing words (i.e., Time on Task) in the Semantic-Other condition, suggesting that the cognitive task was not prioritized. The second is that the differences in number of words produced were accounted for by the proportions used in our analyses, eliminating the possibility that our findings are solely the result of increased word production. As such, these results do not entirely reflect task prioritization or amount of verbal output.

We conclude that the results provide support for our hypothesis that motor-related word production would facilitate motor production (i.e., tapping). Furthermore, we propose that this facilitation is due to the shared neuroanatomical structures that support cognitive and motor processing during language-motor production tasks. That is, the content of what is said can have a rapid influence on a specific body part (i.e., the index finger) when shared semantic-motor representations are engaged.

## Discussion

We examined the effects of shared semantic-motor representations on language and motor production tasks. While previous evidence does support language-motor interaction in a variety of comprehension tasks, the current work elucidates effects of engaging semantic-motor representations on whole body motor tasks (i.e., postural control) and motor tasks requiring dynamic use of a specific body part (i.e., finger tapping). In sum, we were able to demonstrate that engaging shared semantic-motor representations can influence motor performance of the whole body and specific body parts. More specifically, in the context of semantic-motor representations, language production can facilitate motor production and motor production can facilitate language production under dual task conditions.

Experiment 1 demonstrated that generative word production to motor-related categories produced slightly greater COP displacement than generative word production to non-motor related categories. While the results were perhaps influenced by the inequality in number of words produced for one category (i.e., garage tools) in the Semantic-Motor condition, a plausible explanation for the trend observed (i.e., greater COP displacement in the motor-related condition) is that motor word generation is *sufficient* to engage shared semantic-motor representations (i.e., lexical-semantic representations and motor representations). That is, engagement of semantic-motor representations can produce a generalized, low-level effect on the motor system that affects postural control.

Experiment 2 demonstrated that concurrent finger tapping and word production to motor-related categories facilitated performance, as evidenced by the absence of significant dual task effects in the Semantic-Motor condition. This finding extends current research by demonstrating that engaging semantic-motor representations via concurrent language and motor production tasks is *sufficient* to influence word production and motor performance with a specific body part. Thus, engaging semantic-motor representations has a reciprocal effect on language and motor systems when dynamic, self-initiated tasks are completed simultaneously.

These findings support our hypotheses that language and motor systems are functionally linked through shared neuroanatomical structures that support cognitive and motor processing during concurrent, motor-related verbal and manual production tasks. Additionally, by demonstrating that semantic activation of motor-related concepts is *sufficient* to engage the motor system, and that motor activation is *sufficient* to engage the language system, we provide additional support for embodied/modality-specific theories proposing that concepts maintain their sensorimotor states.

Our study was not designed to elucidate the underlying nature of activation in the motor system during semantic processing (i.e., we cannot state that motor activation was due to perceptual enactment), so our evidence does not speak to whether activation of shared semantic-motor representations is both *necessary* and *sufficient* for language and motor production. However, by demonstrating that semantic activation is *sufficient* to engage both systems, we provide additional support for functional language-motor interaction, as well as embodied/modality-specific theories proposing that concepts maintain their sensorimotor states.

When examining broad, high level constructs such as language and motor performance, a number of methodological concerns invariably arise. One of the most obvious potential confounds is a difference in cognitive complexity of the verbal fluency categories. It is possible that production of words in the Semantic-Other conditions was easier because the categories (e.g., animals) were more common and likely represent a more cohesive semantic network than the categories in the Semantic-Motor conditions (e.g., objects that require the use of your hands). A second, related issue is the number of words produced in each category. The Semantic-Other categories (which were presumably less demanding) yielded a greater number of words than the Semantic-Motor condition, thereby increasing concurrent motor activity in that condition. Thus, it is possible that at least part of the observed results reflect a direct or indirect effect of cognitive complexity over an embodied word meaning hypothesis. Furthermore, we cannot definitively state whether cognitive processes exclusive to production of motor-related words contributed to our findings. For example, studies have shown that processing and production of motor words activates cortical structures associated with motor programming through mental simulation [14], [25], a phenomenon not induced in processing of nonmotor (or visual) words. Thus, future studies will benefit from a wider range of verbal fluency categories to parse the potentially confounding effect of cognitive complexity. Additionally, use of neurophysiological measures will help elucidate the underlying nature of motor activation during motor-related production tasks.
